# Cases of Pulmonic Inflammation, Illustrative of Observations on Peripneumony, Inserted in the Medical and Physical Journal for November, 1814

**Published:** 1815-04

**Authors:** W. Robertson


*For the Medical and Physical Journal.*

### *Cases of Pulmonic Inflammation, illustrative of Observations on Peripneumony, inserted in the Medical and Physical Journal for November,* 1814. By W. Robertson, M.D. A.O.L. and C.C.E.

(*Concluded from p.* 178.)

CASE II.—September 10th, 1814. I was requested to visit Dorothy ——, a child of nine years of age, who, five days previously, had, in consequence of exposure to

 cold, cold, been seized with pulmonic complaints. She had been once blooded, and her side was not yet healed from the effects of a blister, ordered by the family surgeon. At the time I saw her, her respiration was performed with much difficulty, and she complained of a sense of weight rather than of pain, which she referred to a point equidistant from the sternal end of the left clavicle and insertion of the cartilage of the last true rib. Her face was much flushed, and there was a circumscribed redness on the cheek, a rawness of the tarsi, a fullness of upper lip, a protuberance and durity of abdomen, which foreboded both present and future evil; yet the eyes were not pearly, there was no night fever, no emaciation, no diarrhœa, and the expectoration excreted by a cough, which was very frequent and harassing, appeared to be frothy mucus only.* The nose and face were swelled, and gave a peculiar apish character to the countenance;† the eyes were heavy, clouded, and devoid of animation; the lips full and dark-coloured, yet dry and scaly. She complained of incessant palpitation; and the pulse was frequent, feeble, and irregular. The skin was irregularly hot and dry, though the face and neck were greasy. The belly had been kept open by clysters, but the tongue was foul. Appetite gone, and she was affected with frequent vomiting after fits of coughing. Sleep also had fled, but the body was round and plump. Fiat fonticulus ex parte vesicata ope unguenti pulveris Cantharidum Injiciatur Clysma statim.

℞. Olei Amygdalæ ℥vi.
Syrupi Tolutani ℥j.
Aquæ distillatæ ℥v. liquoris potassæ subcarbonatis q. s. ad emulsionem formandam.
Sit dosis cochliare magnum urgente tussi.

℞. Liquoris Antimonii tartarizati, Syrupi tolutani āā ℥ſs.

Potassæ Nitratis ℈ij.

Misturæ Camphoræ ℥vi.

Misce sumat cochleare magnum 3tia quaque horâ utatur pediluvio—horâ somni.

Milk diet.

11th. Some moisture of skin from the diaphoretic, and much nausea, but no vomiting. Pulse 120, feeble and irregular. Face much swelled, and in some places livid. Breath-

* It is remarkable that external scrophula generally appears under puberty, and phthysis pulmonis always after it; at least it is a very rare disease in infancy.

† Aretæus mentions this appearance as characteristic of peripneumony.

ing ing not relieved, supposed to be worse. Cough severe, particularly in the night. Expectoration of mucus pretty free, not tinged with blood, but there is some suspicion of pus being mixed with the mucus. Complains much of palpitation, and still more of the issue. To be kept in the erect position, or as near that posture as may be. Continuentur Medicamenta per hunc diem, ut heri præscripta.

12th. No improvement. Breathing more laborious, and expectoration less free. Face and lips more tumid, and the eye-lids nearly approximate from a glazed puffy inflation, while the whole countenance is expressive of extreme dejection and miserable anxiety. She was delirious in the night, but at present is quite collected. Skin not hot, but feels clammy and unctuous to the finger. Pulse 96, and irregular. Is unable to bear the erect posture, reclining now on her back, and she is sunk in the bed. Can take no food. Urine deeply tinged; and the last feculent discharge was clay-coloured. Abdomen less hard, but certainly larger than natural. Omittatur mistura diaphoretica, continuentur alia. To have beef-tea and wine-and-water for food and drink.

℞. Hydrargyri Submuriatis gr. ſs,
Pulveris Antimonialis gr. ij.
Sacchari Albi gr. v,
Misce fiat Pulvis 4tis horis sumendus, et dosibus vespertinis & matutinis adde pulveris Opii gr. ſs.

13th. Has had one copious stool, with much gripes; it is of a green colour, intermixed with gelatinous matter. Some moisture on the skin. Breathing more free, and cough easier. Pulse 100, and irregular. Urine voided in greater quantity, and of a natural colour, or paler. Has slept for some hours. Face less tumid. No appetite. Still some vacillation of mind. Much palpitation. Expectoration freer, though not so abundant as formerly. Continuentur omnia & jusculum bovinum & vinum.

14th. Has taken some beef-tea and wine, Breathing much less laborious. Cough not so troublesome. Less swelling of face, and eyes more animated. Mucous expectoration effected with more facility. Continuentur remedia.

15th. Is considerably improved in every respect. Omittatur jusculum bovinum and vinum.—To have milk diet. Continuentur medicamenta.

16th. Breathing much less laborious. Cough less troublesome, and face more natural. Continuentur medicamenta.

Note.—From this period, the patient's improvement was rapid and progressive. The mercurial medicine was continued for a few days after the pectoral complaints had disappeared; and the patient is now in her usual health. The tarsi tarsi of both eyes are still raw, but are improving under the use of the Unguentum Hydrargyri nitratum.

*Observations.*—In this case of Peripneumony, the subject was imbued with most of the marks of a scrophulous constitution, in which the use of mercury is supposed to be productive of mischievous effects; yet the treatment of this disease of peripneumony by mercury was completetely successful.* There was no relapse, as in the former and subsequent cases, and this is to be attributed to the remedy being continued till the disease was entirely subdued. The complaint, from the beginning, was attended with little thoracic pain. This is very often the case. The older physicians even doubted if the lungs were susceptible of pain. Aretæus writes "simul adest thoracis gravitas, doloris absentia si solus pulmo inflammatur, quippe qui naturaliter doloris immunis sit;"† and Celsus Aurelianus observes, "gravitatio sine ullo dolore, aut cum parvo."‡ I have no doubt that this circumstance, of peripneumony being often unattended by pain, enables us in a certain degree to explain why this disease is considered to be so much less prevalent in this island than on the continent and in America. The fact is, that the complaint is often not attended to, and very frequently misunderstood, merely from the absence of acute pain. Attacks of this disease, when they do not prove fatal, terminate often in phthysis pulmonalis§ in the young, and in hydrothorax‖ in those more advanced in life. On the prevalency of these maladies, it were worse than useless to expatiate.

---

* The difficulty of curing lues in scrophulous habits has tended much to confirm this opinion,—an opinion which, in many instances, might be controverted.

† De causis et signis Morb. Acut, p. 10.

‡ P. 139, lib. ii. cap. 27. See also Aëtius, tetrab. ii. serm. 4, p. 518; Trallian, lib. v. p. 241; Ægineta, lib. iii. p. 40; Celsus, p. 224. See also Van Swieton, who quotes the above authorities, vol. viii. p. 199.

§ I know that it is doubted if regular phthysis ever be produced by peripneumony, but in scrophulous constitutions, I presume, it will be admitted as a powerful exciting cause. The third case of peripneumony in this paper indubitably terminated in confirmed phthysis pulmonalis.

‖ The hydropic condition has certainly some analogy to the phlogistic, in as far as increased exhalation takes place. That the latter state passes into the former occasionally, in all the cavities of the body, we have no doubt; and we sometimes witness the same on the surface, particularly in that species of inflammation termed erythematic.

There

There was some suspicion of purulent matter being excreted from the lungs in this case, in considerable quantity, but the reporter had no opportunity of ascertaining this circumstance, the suspected matter having been carelessly put aside, and no similar expectoration recurring. It appeared to the reporter, from an attentive consideration of many of the symptoms, that effusion to a certain amount had taken place previously to his being consulted; but, if such did occur, the effused fluid was probably reabsorbed. The flushing of the face was much circumscribed, as in hectic; and the eyes had from the beginning a more heavy and clouded aspect than is generally witnessed even in this disease. The cornea also showed a peculiarly dull greasy appearance. All these symptoms are well marked by Ægineta, who says, "Genæ itaque in his rubræ apparent, & occuli intumescunt, supercilia deorsum nutant, & corneæ subpingues apparent." *Lib.* iii. p. 40.

On the whole, I considered the case as a very critical one, and gave an unfavourable prognosis, but was happily disappointed in the termination. There appeared to be no deficiency in the conformation of the chest; but the scrophulous habit was indicated so decidedly, that I dreaded the collision of two such diseases in so tender a subject.

Case III. (*Peripneumonia Notha.*)—March 4th, 1814. I was consulted for Mr. ——, ætatis 67, whom I knew to have been intemperate in the use of fermented liquors during many years. But, though advanced in life, he had not previously been subjected to severe indispositions. I learnt from his family that he had been confined to bed during four days, in consequence of having been seized on the first of this month, after a fit of intoxication, with shiverings alternating with hot fits, which were succeeded by cough, pain of the left side, and very considerable difficulty of breathing. I found him in the above state, with his head and shoulders elevated on pillows, and the window of his room thrown open. He complained more of the difficulty of breathing, than of the pain. Pulse 96, with an intermission after every fourth or fifth stroke. Cough incessant, with much sputum, not tinged with blood. Thirst considerable. Tongue foul and slimy. Urine high-coloured, and in small quantity. Face tumid, and of a purple colour, though his intemperance may in part have occasioned this livid colour. Lungs cannot be inflated beyond a certain point, in consequence of pain between the seventh and eighth ribs. General expression of countenance and attitude of body indicative of extreme irritability, old intemperance, much anxiety and oppression, great prostration of the moving powers, and morbid venous accumulation accumulation in the head and neck. Voice accords, feeble, irritable, and querulous. His age, habits, and long residence in a warm climate, have rendered his system incapable of tolerating either his disease or the remedies; the continuance of the former, and the adoption of the latter, seem to tend towards the same point; forming one of those perplexing cases of contra-indications which occasionally occur in practice. Appetite and sleep gone. Bowels costive. Refuses a clyster.

℞. Submuriatis Hydrargyri gr. iij.
Pulveris Jalapii gr. viij.
Olei Menthæ gtt. ij. fiat Bolus statim capiendus.
Applicetur lateri, Emplastrum Epispasticum satis amplum.
Mittatur sanguis ê brachio, (pleno rivo) ad ℥xij.
Utatur Mistura Mucilaginosa, urgente tussi, & Decocto hordei ad libitum.

5th.—A firm buffy coat on the blood. Breathing somewhat relieved, and the pain of side not felt. Blister produced no effect. Thirst urgent. Face grim and clouded. Eye-lids inflated, smooth and shining. Skin in colour, ochreous and dingy; to the finger clammy and unctuous. Body sunk, with the feet beyond the bed-frame. Cough incessant. One dejection from the bolus. Mittatur sanguis iterum e brachio ad ℥viij. nisi prius supervenerit syncope. (5th day of the disease). Repetatur etiam Emplastrum Cantharidis, Pulvere Meloës bene aspergendum.

℞. Liquoris Ammoniæ Acetatis ℥iij.

Aquæ Menthæ ℥x.

Syrupi ℥j.

Liquoris Antimonii tartarizati gtt. xv. fiat haustus 3tia quaque hora sumendus.

6th. Blood in the first cup covered with a buffy coat; the second not so. Breathing, cough, and pulse, as yesterday. Much nausea, but no general moisture. Blister has risen well. Face and throat more tumid, and he raves occasionally. Skin tender when touched. Continuentur Medicamenta & horâ somni utatur pediluvio.

7th. Breathing very difficult, and the sputa excreted with much exhausting labour. Face more tumid and livid. Lips blue. Eyes sunk in their orbits. Pulse 90, feeble and intermitting. Has been threatened with suffocation at different times during the day. Is now in the half-erect posture. Says "his breath is about to leave him." Anxiety and restlessness distressing. Heat below the natural standard. Moving powers nearly exhausted; and a general collapse of the system seems fast approaching, while the lungs appear to be deluged with with blood, and their functions nearly extinct. Habeat Vini ℥x. Capiat partitis vicibus, Jusculum Bovinum, ad libitum. Applicetur Emplastrum Cantharidis inter scapulas, & Cataplasma (ex pulvere sinapeos, &c.) pedibus, more solito. Omittatur Mistura diaphoretica.

℞. Hydrargyri Submuriatis gr. i.
Pulveris Antimonialis gr. iij.
Pulveris Opii gr. ſs.
Capiat statim & Repetatur secunda quaque hora ad quartam vicem, ex syrupo papaveris albi.

To be kept in the half-erect posture. To have a pint of warm porter with sugar, at his own desire.

8th. Reaction commences apace, and the centre is relieved. A general moisture covers the whole body. Breathing less laborious. Pulse 100, and irregular, but stronger. Expectorates much bloody mucus. Cough incessant. Face livid and swollen. No stool. Tongue slimy. Intermittatur Vinum. To have a pint of warm water, with sugar, three times a-day. Continuatur Pulvis 4ta quaque horâ & Mistura Mucilaginosa & Jusculum Bovinum.

9th. Considerable amendment. Pulse 96, full, but irregular. Face less swollen and livid. Eyes more animated. Skin soft and moist, still tender to the touch. Urine copious. Cough very troublesome and exhausting. Breathing considerably improved. Sputa in small quantity. Continuatur pulvis mane & nocte & alia. Intermittatur Cerevisia & Jusculum Bovinum. Milk diet.

10th. Is much improved, though the cough is distressing. Breathing easier. Pulse 96, with intermissions. Refuses to take any more medicine. Strength so much improved, that he is sitting by a fire, and he has some appetite. Insists on his porter being continued, and he requests to have wine. Intermittatur & Vinum & Cerevisia & Jusculum Bovinum.

Note.—In consequence of the peculiar disposition of the patient, the reporter ceased in a great measure his attendance, the medicines being discontinued. He, however, understood, that four days subsequent to the last report, the patient was seized with acute pain in the left side, rapid and regular pulse, augmentation of cough and fever, constituting that species of pulmonic disease termed by Cullen Pneumonia (pleuritis), but which might, perhaps, be more appropriately designated Pulmonitis. This disease, in despite of the usual remedies, terminated on the ninth day from its commencement, in suppuration, preceded by a cessation of pain, shiverings, &c. and purulent matter, to the amount of four or five ounces, was expectorated at one time, after a severe fit of coughing, which had nearly suffocated the un-

 happy happy man. Four months subsequent to this period, the reporter was hastily summoned to visit the patient, who was found surrounded by his weeping family. His face, now pale and ghastly, was so much swollen, that not a feature could be recognised. His eyes were closed. His mouth covered with apthæ. His beard matted with pus; and from the lowest corner of his mouth flowed a stream of the same matter, which moistened his pillow. Colliquative sweats and diarrhœa had extenuated his withered body; and the few white hairs of his forehead had dropped. His nails were adunque. The angles and processes of the bones shone through the nearly diaphanous skin, which seemed suspended from point to point. His chest panted for breath. The pulse at the arm was not to be felt, yet the heart continued to beat. In a few hours thereafter he expired.

I had intended to have offered some remarks on Peripneumonia Notha, so denominated by Sydenham, and adopted by Boerhaave (though under this title they describe diseases quite dissimilar); but the great extent to which this paper has reached, precludes my encroaching further at this time on your valuable pages.

*Berwick-on-Tweed;* W. ROBERTSON.

*Dec.* 28, 1814.

---

*For the Medical and Physical Journal.*

